# Advances in Ceramic–Carbonate Dual-Phase Membrane Reactors for Direct CO_2_ Separation and Utilization

**DOI:** 10.3390/membranes15020053

**Published:** 2025-02-06

**Authors:** Xue Kang, Qing Yang, Jiajie Ma, Qiangchao Sun, Hongwei Cheng

**Affiliations:** 1 Department of Chemical and Material Engineering, Lyuliang University, Lvliang 033001, China; 20201016@llu.edu.cn; 2 School of Materials Science and Engineering & State Key Laboratory of Advanced Special Steel, Shanghai University, Shanghai 200444, China; 24722465@shu.edu.cn (Q.Y.); majj1638981894@shu.edu.cn (J.M.); tonysun@shu.edu.cn (Q.S.)

**Keywords:** CO_2_ separation, post-combustion, ceramic–carbonate dual-phase membranes membrane reactor, mixed ionic–electronic conducting

## Abstract

Excessive (carbon dioxide) CO_2_ emissions are a primary factor contributing to climate change. As one of the crucial technologies for alleviating CO_2_ emissions, carbon capture and utilization (CCU) technology has attracted considerable global attention. Technologies for capturing CO_2_ in extreme circumstances are indispensable for regulating CO_2_ levels in industrial processes. The unique separation characteristics of the ceramic–carbonate dual-phase (CCDP) membranes are increasingly employed for CO_2_ separation at high temperatures due to their outstanding chemical, thermal durability, and mechanical strength. This paper presents an overview of CO_2_ capture approaches and materials. It also elaborates on the research progress of three types of CCDP membranes with distinct permeation mechanisms, concentrating on their principles, materials, and structures. Additionally, several typical membrane reactors, such as the dry reforming of methane (DRM) and reverse water–gas shift (RWGS), are discussed to demonstrate how captured CO_2_ can function as a soft oxidant, converting feedstocks into valuable products through oxidation pathways designed within a single reactor. Finally, the future challenges and prospects of high-temperature CCDP membrane technologies and their related reactors are proposed.

## 1. Introduction

**Figure 1:**
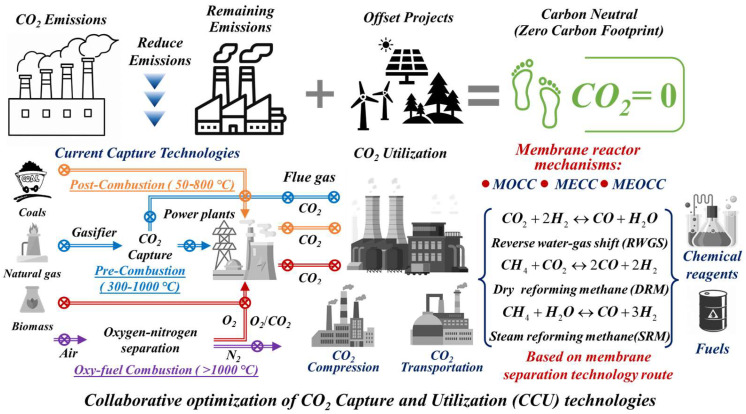
A schematic summary of the main components in this review. Global carbon neutral pledge-related projects and pathways, CO_2_ capture and utilization technologies, and membrane reactor-based materials for post-combustion technology.

The rapid growth of natural activities (such as volcanic activity and changes in the World Ocean, etc.) and industrial production on a global scale has put carbon dioxide (CO_2_) emissions at unprecedented levels (407.8 ppm, 2023). It is expected to reach 422.5 ppm, and the CO_2_ emissions from fossil fuel combustion will increase to 3.8 Gt in 2030, resulting in an increase in seawater level and global warming [1,2]. The Intergovernmental Panel on Climate Change (IPCC) has indicated that human activities account for 76% of climate change, while natural factors contribute only 24%, with the proportion of human influence continuing to rise. Natural processes such as volcanic activity, ocean–atmosphere exchanges, and plant respiration contribute to the carbon cycle, with these processes generally remaining in balance. However, human activities, including the burning of fossil fuels, deforestation, and industrial production, account for more than 75% of global CO_2_ emissions annually. Although global countries have promulgated carbon neutrality pledges and implemented offset projects (e.g., the usage of renewable energy, improving the efficiency of current energy utilization, etc.) to achieve net-zero carbon footprints (Figure 1, the upper part), the large-scale application of energy storage technology and the maturity of advanced turbine technology cannot be achieved in a short time [3,4,5,6]. To this end, direct CO_2_ capture from the existing power plants, such as flue gas, a major emitting source, is regarded as a promising solution to mitigate climate change caused by CO_2_ emissions [7]. Moreover, the former CO_2_ capture from industrial flue gas can be coupled with the subsequent utilization to prepare value-added chemical products (methanol, syngas, etc.), and this so-called carbon capture and utilization (CCU) technology [8,9,10].

Reaching zero carbon emissions by 2060 in China requires rapid actions in both CO_2_ emission reductions from the large emitting sources by applying CCU technology and switching to sustainable energy systems step by step [11,12,13,14]. Three technology routes at an industrial scale have been developed for CO_2_ capture from coal-derived power generation: post-combustion [15,16], pre-combustion [17,18], and oxy-fuel combustion [19,20], as exhibited in Figure 1 (bottom left part). In general, post-combustion capture applies primarily to coal-fueled air-fired power plants. Pre-combustion capture applies to gasification plants. In comparison, oxy-fuel combustion capture can be applied to new generators or retrofitted to existing plants. Readers can find more details on the above three CO_2_ capture technologies in other reviews [21,22,23]. Among these CO_2_ capture technologies, post-combustion CO_2_ capture approaches (i.e., chemical absorption [24], adsorption [25], cryogenic distillation [26], and membrane-based separation [27,28,29]) are the greatest near-term potential for reducing CO_2_ emissions, because they can be retrofitted to existing units that generate two-thirds of the CO_2_ emissions in the power plants. Among them, membrane-based separations can be cost-effective in terms of operational time durations, temperature, pressure, and energy requirements as compared to separations based on absorption/adsorption processes [30]. The main candidate materials for membranes are polymer, inorganic membranes, and mixed-matrix membranes. However, the stability of these polymer membranes under actual operation conditions remains rather poor, and the permeability–selectivity “tradeoff” effects are still a matter of concern, particularly at high temperatures [31]. Moreover, most of them are operated at relatively low temperatures (<300 °C), which is not suitable for post-combustion CO_2_ capture from flue gas at high temperature conditions (Figure 1, bottom middle part) [32,33].

Microporous inorganic membranes are widely used and remain stable at elevated temperatures; however, their performance is compromised due to transportation mechanisms such as sintering and molecular sieving at high temperatures. Among the various classes of membrane materials that have been reported, one category of membranes suitable for overcoming the above challenges is the ceramic–carbonate dual-phase (CCDP) membrane [34,35]. As an emerging high-temperature CO_2_ separation material, the CCDP membranes have attracted considerable attention for their application in high-temperature CO_2_ separation, considering their superior chemical, thermal stability, and mechanical strength [36,37]. Moreover, the application of the CCDP membrane is not limited to CO_2_ separation. It can also be integrated into a catalytic membrane reactor for CO_2_ recovery from flue gas into a highly valuable chemical products (e.g., syngas, methanol, etc., Figure 1, bottom right part) in the single reactor through reforming reactions, such as reverse water–gas shift (RWGS), the dry reforming of methane (DRM), and the steam reforming of methane (SRM) [38,39,40].

Owing to the relatively brief history of the development of the CCDP membranes, there are merely a few review articles offering a comprehensive review of the CCDP membranes, mainly discussing the synthesis methods, CO_2_ permeation mechanisms, and permeation properties of the CCDP membranes. More significantly, the research groups of Fu, Terry, and Zhang have consistently highlighted the crucial factors and challenges that affect the synthesis and application of the CCDP membranes for high-temperature CO_2_ capture and utilization [36,41,42]. Therefore, it is necessary and timely to provide a comprehensive summary of the CCDP membranes for CCUS technology.

In this context, this review initiates with the fundamental principle of CO_2_ capture chemistry and the transport theory of three types of membrane reactors. Subsequently, a meticulous assessment is conducted on how the design materials and their inherent attributes, along with the surface modification, contribute to CO_2_ separation and utilization. Significantly, several typical membrane reactor concepts are subsequently presented to illustrate how the captured CO_2_ acts as a soft oxidizer to transform feedstocks into valuable products through the oxidative route in single-reactor designs. Ultimately, the future development, challenges, and prospects of high-temperature CCDP-based membrane technology and the related reactors are candidly deliberated.

## 2. Post-Combustion Decarbonization Technology

### 2.1. Typical Four Post-Combustion CO_2_ Capture Approaches

**Figure 2:**
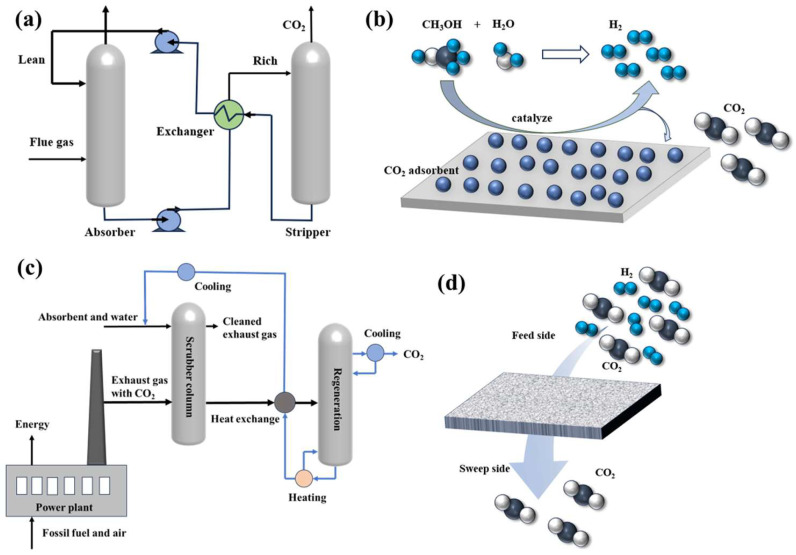
(**a**) Flow sheet of the chemical absorption. (**b**) Mechanism diagram of adsorption. (**c**) Flow sheet of the cryogenic distillation. (**d**) Mechanism diagram of the membrane separation method.

Up to now, the most widely used post-combustion decarbonization technology mainly included chemical absorption, adsorption, cryogenic distillation, and membrane separation technology (as illustrated in Figure 2 and Table 1) [29]. Chemical absorption is a relatively mature CO_2_ capture approach, using an amine-based solvent based on the reversible chemical absorption principle to absorb CO_2_ into the bulk phase of another material to achieve CO_2_ enrichment. The flow chart of the common chemical absorption process is shown in Figure 2a. The solutions, rich in CO_2,_ are dispatched to the resolver (or stripping tower), and the CO_2_ within the absorber is liberated through heating to achieve the objective of recycling [43]. However, drawbacks like the toxicity and degradation of the solvent, the high energy consumption in solvent regeneration, the corrosion, and environmental concerns regarding solvent disposal have rendered its applications [44]. Currently, researchers are endeavoring to overcome these challenges. For instance, Sanz-Perez et al. employed spray towers instead of packed towers to lower the cost of large, packed towers [45]. Zheng et al. installed a heat exchanger to reduce energy consumption [43].

Adsorption is a methodology through weak Van der Waals forces (physical adsorption) or strong covalent bond forces (chemisorption) to achieve CO_2_ enrichment (Figure 2b). The adsorption takes advantage of the adsorption of CO_2_ by the adsorbent for the separation of CO_2_, while the absorption makes use of the high solubility of CO_2_ in a specific solvent [46]. The adsorption approach boasts the merits of energy saving, low costs, and simplicity in equipment and separation processes. However, its development is constrained by the low adsorption capacity and the difficulties in the regeneration of adsorbents [21].

Cryogenic distillation is primarily founded on the physical properties of CO_2_, and the flue gas is compressed several times, and then liquefied by low-temperature treatment to achieve separation [47]. As shown in Figure 2c, the flue gas is compressed multiple times and subsequently liquefied through low-temperature treatment to achieve separation. Its advantage lies in the absence of chemical absorbents, being applicable at atmospheric pressures, and the ability to produce liquid CO_2_ for pipeline transportation [48]. Nevertheless, it demands refrigeration energy, and the separation effect is suboptimal, restricting the application scope of CO_2_ capture.

In contrast to other separation approaches with high energy consumption, membrane separation technology is characterized by its continuity, cost-effectiveness, energy efficiency, and ability to handle gasses at high flow rates, thereby rendering it particularly appropriate for CO_2_ capture [49]. The development of membrane separation technology has witnessed significant progress over the years, as evidenced by a growing body of research conducted globally. The working principle is shown in Figure 2d. There are many kinds of membranes for CO_2_ capture, such as organic polymer membranes, inorganic porous and dense membranes, etc. Therefore, in the following content, we will be focusing on the membrane reactor technology for CO_2_ separation and utilization, from the membrane materials category to the mechanisms of the CCDP-based membrane and its applications.

**Table 1:** The comparison of three post-combustion CO_2_ capture methods in terms of mechanisms, advantages, disadvantages, and main materials.

Method	Mechanisms	Advantages	Disadvantages	Materials	Ref.
Chemical absorption	Absorb CO_2_ into the bulk phase of another to achieve CO_2_ enrichment.	Established technology for large applications	High energy consumption, increasing costs, and deterioration in long-term performance	Alcohol amine solutions; alkali metal salt solutions	[44,50]
Adsorption	Selectively absorbing CO_2_ onto the surface of another material through weak Van der Waals forces or strong covalent bond forces.	Low energy consumption, simplicity in equipment and separation processes	Low adsorption capacity, difficulties in regeneration of adsorbents	Carbon; metal oxides	[21,46]
Cryogenic distillation	Separate CO_2_ through cryogenic liquefaction.	No chemical absorbents, available at atmospheric pressures	Costly heat transfer; high energy consumption to refrigeration	Cryogenic distillation	[48]
Membrane technology	Capture CO_2_ by differences in the solubility or diffusivity of it on both sides of the separated membrane.	Low energy consumption, low costs, environmentally friendly	Membrane plasticization and degradation, mutual constraint between permeability and selectivity	Polymer membranes; inorganic membranes	[49,51,52,53]

### 2.2. Membrane-Based Post-Combustion CO_2_ Capture Technology

The membrane types that are generally used for low-temperature CO_2_ separation (<300 °C) are polymeric and inorganic microporous, while the ceramic–carbonate dual-phase membranes are usually employed at high-temperature CO_2_ separation (550–950 °C) [54,55,56,57]. The major challenges faced by the membrane separation technology lie in achieving high permeability, selectivity, prolonged operational hours, and stability at elevated temperatures. They will be discussed in detail in the following two sections, respectively.

#### 2.2.1. Low-Temperature Membrane Technology

**Figure 3:**
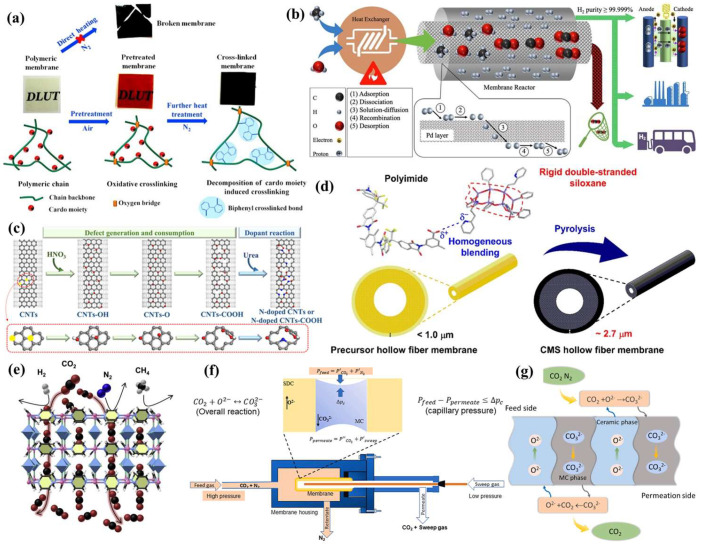
Membrane separation technology. (**a**) The preparation scheme of the crosslinked polymer membrane [58]. (**b**) The palladium-based metal membrane for H_2_ separation [60]. (**c**) The activation mechanism of the N-doped CNTs membrane [61]. (**d**) The illustration of the homogeneous hollow-fiber membranes [62]. (**e**) The structure of a metal–organic framework membrane [64]. (**f**) The schematic drawing of the setup and CCDP membrane for CO_2_ permeation [67]. (**g**) The schematic illustration of the CCDP membrane for CO_2_ separation [68].

Organic polymer membranes based on the solution–diffusion mechanism were the earliest reported gas separation membranes and were frequently developed for CO_2_ separation from natural gas and flue gas [31]. Nevertheless, their inadequate thermodynamic and chemical stability, as well as their low permeability and selectivity, restricted their wide applications, which is mainly attributed to their plasticization and physical aging during their long-term operational life. Recently, Xu et al. prepared a thermal crosslinked film with high plasticization resistance and high gas selectivity for CO_2_ separation (as displayed in Figure 3a) [58]. Very recently, Liang and co-authors improved the gas separation performance of polyether block amide membranes by blending polydimethylsiloxane-polyethylene oxide block copolymer [59]. Balancing the “trade-off” effect between permeability and selectivity in organic separation membranes is a crucial step in improving their level of CO_2_ separation efficiency.

Another type consists of inorganic membrane materials, which are typically composed of metals or oxides and classified into porous and dense membranes. In contrast to polymer membranes, inorganic membranes possess the advantages of superior chemical stability, high mechanical and thermal stability, adjustable pore size distribution, and less plasticization. In 2021, Kian and co-authors fabricated a palladium-based metal membrane reactor for the separation of H_2_ and have further comprehended the development prospect of CO_2_ separation and utilization (Figure 3b) [60].

A typical carbon membrane is fabricated from materials like activated carbon or silicon carbide, which undergo special treatment to form a carbon membrane featuring a uniform pore size distribution for the efficient separation of CO_2_. In 2023, Gao et al. prepared an N-doped carbon nanotubes (CNTs) membrane with the -COOH functional group to facilitate the rate of CO_2_ adsorption and resolution process [61], as illustrated in Figure 3c. As shown in Figure 3d, Shin and co-authors prepared a high-performance carbon molecular sieve hollow-fiber membrane by the pyrolysis of rigid double-chain siloxane precursors for CO_2_ capture in 2019 [62]. The metal–organic framework (MOF)-derived membranes constitute a novel class of materials that form highly regular porous networks through the connection of metal ions or clusters with organic junctions [63]. Owing to its regular structure, high surface area, adjustable pore size and the like, it has attracted considerable attention. In 2023, as depicted in Figure 3e, Zhou and his co-authors developed a recognition MOF membrane via a “steam-assisted surface recombination” strategy for efficient CO_2_ capture. Notably, the membranes have the advantage of higher permeability [64].

#### 2.2.2. High-Temperature Membrane Technology

To overcome the operating conditions in practical applications, membrane reactors frequently encounter requirements such as high temperatures, long-term operational stability, etc. Moreover, due to the CO_2_ concentration of flue gas being less than 15%, the thermodynamic driving force for CO_2_ capture from flue gas is low, which creates a critical challenge for developing a cost-efficient solution in achieving high CO_2_ capture levels [65,66]. Therefore, this section will primarily elaborate on the ceramic–carbonate dual-phase (CCDP) membranes that can be employed for high-temperature CO_2_ separation. In a CCDP membrane, the typical separation mechanism is that the CO_2_ reacts with O^2−^ from the gas mixture to form CO_3_^2−^ in a ceramic scaffold, while the CO_3_^2−^ is transported in molten carbonate and decomposes into CO_2_ and O^2−^ on the purge side of the membrane, while O^2−^ migrates through the ceramic phase to form a closed cycle [37].

In recent years, a considerable number of fundamental investigations regarding the types of carbonates, ceramic phase compositions, and microstructures have been presented. Ovalle-Encinia et al. investigated the CO_2_ permeation flux of the dual-phase samarium-doped ceria (SDC) and molten-carbonate (MC) membranes and the stability of molten carbonate under high pressure (Figure 3f). The CO_2_ stability of the membrane reactor was significantly improved due to the better mechanical properties of the support with less than 7% porosity [67]. In Figure 3g, the Wu group designed a Ce_0.8_Nd_0.2_O_2−δ_ (NDC) carbonate hollow-fiber membrane and tested its permeability behavior and thermal and chemical stability during the high-temperature CO_2_ separation. The improvement mechanism of this design has been comprehensively characterized by employing multi-dimensional test profiling [68].

### 2.3. The CO_2_ Permeation Chemistry of the CCDP Membrane

The CCDP CO_2_-permeable membranes are composed of one (or two) porous solid matrix filtered with a molten-carbonate (MC) phase. The solid matrices are porous metals or oxide–ionic-conducting ceramics, providing the requisite electronic and/or oxide ionic conduction and charge compensation for the MC phase [69,70]. The latter acts as a carbonate-ionic conductor and a sealant to render the membrane gastight. Thus, when making selections and preparations for matrix materials, the ensuing prerequisites must be adhered to (1) oxide ionic, or electronic, or mixed oxide ionic and electronic conductor; (2) good chemical compatibility and wettability with MC phase; (3) high chemical stability at high temperatures in CO_2_-containing gasses; (4) excellent mechanical strength [71].

Generally, the CCDP membranes can be categorized into three types according to the carriers of the ceramic phase, which are mixed electron and carbonate-ion-conducting (MECC), mixed oxygen and carbonate-ion-conducting (MOCC), and mixed electron, oxygen, and carbonate-ion-conducting (MEOCC) membranes. In contrast to low-temperature membranes (Section 2.2.1), the CO_2_ that permeates through the CCDP-type membranes is mainly governed by ionic and electronic transport instead of molecular size and charge.

**Figure 4:**
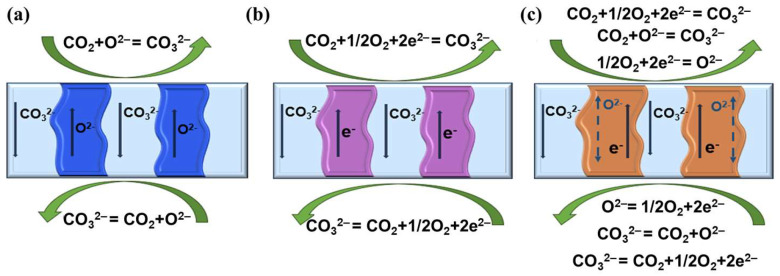
Schematic diagram of CO_2_ separation mechanism in (**a**) MOCC, (**b**) MECC, and (**c**) MEOCC.

As depicted in Figure 4b, within the MECC membranes, the gaseous CO_2_ on the feed side reacts with electrons from the porous support phase and O_2_, thereby converting into CO_3_^2−^ anions. Subsequently, the CO_3_^2−^ anions are transported from the feed side to the permeate side via the carbonate phase. Consequently, the CO_3_^2−^ anions reconstitute into gaseous CO_2_ at the permeate side by releasing the electrons back into the metal phase [72,73]. In the MOCC membranes, the gaseous CO_2_ at the feed side reacts with O^2−^, which is transported from the ceramic porous support phase to form CO_3_^2−^ anions. The CO_3_^2−^ anion is transported from the feed side to the permeate side through the carbonate phase, where it reacts to produce O^2−^ and CO_2_. The O^2−^ ion then returns to the feed side as a compensating ion (as shown in Figure 4a) [74,75,76]. In contrast to the transmission mechanisms of the MECC and MOCC, the MEOCC membranes demonstrate both mixed electron and carbonate ion conduction (as illustrated in Figure 4c). Therefore, this type of membrane can also function in the presence of O_2_. Moreover, the permeation of CO_2_ can be further augmented as more CO_3_^2−^ anions can be produced from the reactions with the O^2−^ anions released from the ceramic phase and the fed O_2_ gas [41,77,78].

## 3. Material Designing and Performance Optimizing

Considering the fundamental perceptions derived from the diverse mechanisms, within this section, our primary focus lies in the effects of material design, microstructure engineering, and operating conditions on CO_2_ permeability as well as long-term durability. Moreover, research indicates that MC phases show less influence on CO_2_ permeability than the oxide ion conductor phase for the CCDP-based membrane materials. Thereby, the main research interests on CO_2_-permeable membranes are focused on matrix materials according to the different permeating mechanisms. Studies on the three mechanisms in recent years are also listed in Table 2, Table 3 and Table 4.

### 3.1. MOCCs-Based Membrane Materials

Fluorite materials with an AO_2_-type structure, including rare-earth ion-doped ceria and yttria-stabilized zirconia (YSZ) [79,80], demonstrate high O^2−^ conductivity and have been extensively studied as porous ceramic supports for MOCC membranes [81,82]. A major challenge with these membranes is their limited chemical compatibility and stability at high temperatures [42,83,84]. MOCC membranes undergo electrochemical reactions at the three-phase interface involving carbonate, the ceramic framework, and the gas phase, as illustrated in Equation (1). Alternative reaction pathways may also occur (see Equations (2) and (3)) [85]. In these reactions, CO_2_ preferentially reacts with CO_3_^2−^ at the two-phase interface to form C_2_O_5_^2−^, which then diffuses through the bulk and reacts with O^2−^ on the opposite side to regenerate CO_3_^2−^.

(1) $$CO_{2}+O^{2-}=CO_{3}^{2-}$$

(2) $$CO_{2}+CO_{3}^{2-}=C_{2}O_{3}^{2-}$$

(3) $$C_{2}O_{3}^{2-}+O^{2-}=CO_{3}^{2-}$$

During this reaction, the partial pressure gradient of CO_2_ serves as the driving force. The gas diffusion flux is commonly described by the Wagner equation [86], which is based on classical diffusion theory.

(4) $$J_{CO_{2}}=\int_{P_{CO_{2}^{\prime \prime }}}^{P_{CO_{2}^{\prime }}}\frac{kRT}{4F^{2}L}d\ln(P_{CO_{2}})J_{i}=-\frac{\sigma_{i}}{{\left(z_{i}F\right)}^{2}}(\Delta \mu_{i}-z_{i}f\Delta \phi )$$

*i* represents the transported species; *J* is the permeation rate; *σ_i_* and *z_i_* represent the concentration, conductivity, and charge number of species *i*, respectively. *μ*_*i*_ and *ϕ* represent the chemical potential and electrostatic potential, respectively; Δ denotes the gradient; *R*, *F*, and *T* represent the gas constant, Faraday constant, and temperature, respectively.

For the MOCC membranes, the conductivity of O^2−^ is significantly lower than that of CO_3_^2−^, which makes the mobility of O^2−^ the limiting step in gas transport [70,87,88]. The schematic diagram of MOCC is shown in Figure 4a. Fluorite phases, known for their high oxygen-ion conductivity, have been extensively studied, including YSZ [80], Sm-doped CeO_2_ (SDC), and scandium- and cerium-stabilized zirconia (ScCeSZ) [56]. CeO_2−*δ*_ exhibits excellent chemical stability; however, it has relatively low conductivity, typically around 10^−4^ S cm^−1^.

**Figure 5:**
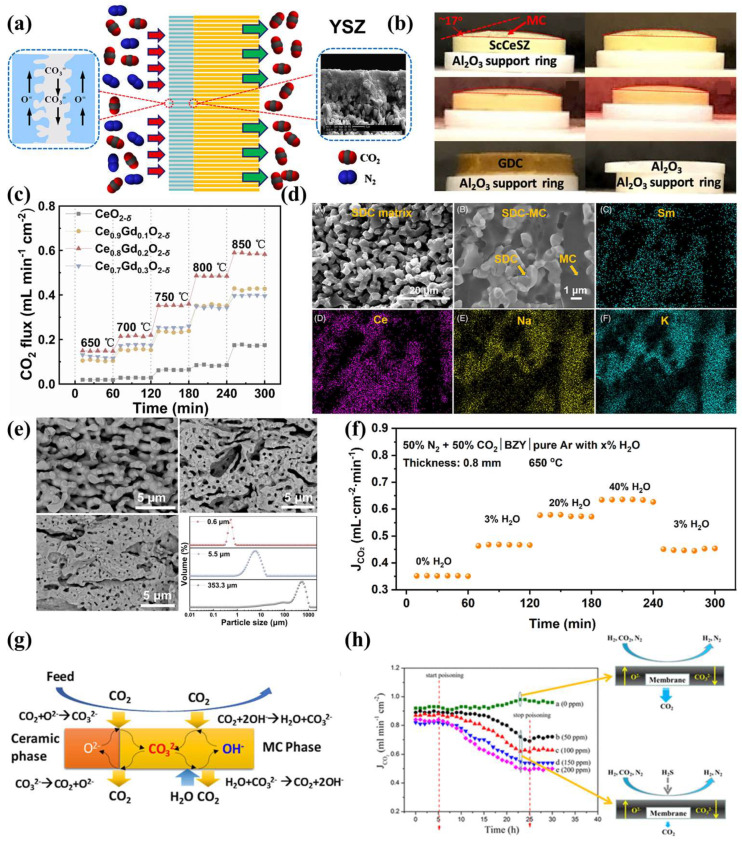
(**a**) Schematic diagram of penetration of YSZ [80]. (**b**) Views of MC on the dense ScCeSZ pellet at a different temperature, reproduced from Ref. [56] with permission from the American Chemical Society, 2021. (**c**) The CO_2_ permeability of Ce_1−*x*_Gd*_x_*O_2−*δ*_-MC (*x* = 0.00–0.30) dual-phase membranes at different temperatures [79]. (**d**) SEM-EDS image of SDC matrix and SDC-MC membrane [89]. (**e**) SEM of Ce_0.8_Gd_0.2_O_2−*δ*_ supports prepared using pore formers with different particle sizes, and the particle size distribution of the pore former [79]. (**f**) CO_2_ flux of BZY-20C-MC membrane with different H_2_O partial pressure [98]. (**g**) H_2_O-enhanced CO_2_ transport mechanism in the CCDP membranes under wet sweeping gas condition, reproduced from Ref. [56] with permission from the American Chemical Society, 2021. (**h**) CO_2_ permeation stability of SDC–carbonate membranes with H_2_/CO_2_/N_2_ feed containing various concentrations of H_2_S at 750 °C [99].

Doping with divalent and trivalent cations to substitute Ce^4+^ creates additional oxygen vacancies to maintain charge balance, thereby enhancing oxygen-ion conductivity, as demonstrated in Gd-doped CeO_2_ (GDC) [79] and SDC [89]. Gas permeability is influenced not only by the material but also by several factors, such as the size of the pore former particles, membrane thickness, and the gas composition on both sides. These factors have been extensively studied [38,82,83,90,91,92,93,94,95,96,97]. Lu et al. investigated the advantages of asymmetric membranes concerning permeability, focusing on the development of thinner, stronger membrane films [80]. They fabricated a thin YSZ layer on BYS, with YSZ impregnated with carbonate (MC). However, due to the poor wettability between BYS and MC, the carbonate impregnation was effectively inhibited, as illustrated in Figure 5a. High-temperature performance tests at 650 °C showed that the permeation flux reached 3.9 × 10^−3^ mol m^−2^ s^−1^, indicating excellent permeability. This enhancement is attributed to the shorter ion transport distance in thinner membrane films. In Figure 5b, Sun et al. developed a novel CCDP membrane by impregnating scandium- and cerium-stabilized zirconia (ScCeSZ) with carbonate [56]. The study found that ScCeSZ demonstrates high oxygen-ion conductivity, but it has poor wettability with MC, resulting in nitrogen leakage under high-temperature conditions. Then, atomic layer deposition was employed to modify the porous support with a layer of Al_2_O_3_. Under conditions of 650 °C with a 50% CO_2_/50% N_2_ mixture, the permeation flux reached 1.0 mL cm^−2^ min^−1^. Thereby, a suitable Al_2_O_3_ surface coating improves the wettability between the substrate and MC. Additionally, increasing the partial pressure and introducing an appropriate amount of H_2_O can enhance CO_2_ permeation.

In 2024, our group doped various amounts of Gd^3+^ into CeO_2_ and observed that Gd^3+^ doping facilitates the conversion of Ce^4+^ to Ce^3+^, with the highest percentage of Ce^3+^ occurring at a doping level of 0.2 [79]. At 850 °C, the permeation flux reached 0.58 mL cm^−2^ min^−1^ at a doping level of 0.2, which was more than double the previous value. Furthermore, we examined the effect of pore formers with varying particle sizes on ceramic phase porosity, as shown in Figure 5c,e. By selecting pore formers with different particle sizes as sacrificial materials, we synthesized GDC porous support with varying pore structures. The support formed using the smallest particle size exhibited the highest porosity. It is important to note that the CO_2_ permeation flux increased to 0.75 mL cm^−2^ min^−1^. This enhancement was attributed to the high porosity of the porous support, which increased the carbonate loading and further enhanced the gas–liquid interface area and the three-phase boundary length on both the feed and permeate sides. Figure 5d shows the morphology of Sm-doped CeO_2_, where the ceramic phase has a porous structure, which becomes denser after carbonate impregnation, preventing N_2_ leakage. The gas composition on both sides of the membrane plays a critical role in gas permeation [89]. Zhang et al. compared the effects of dry and humid environments on CO_2_ permeation. They observed an interesting phenomenon: as the *P*_H2O_ increased, the CO_2_ permeation flux significantly increased. As shown in Figure 5f, when H_2_O reached 40%, the permeation flux increased to 0.65 mL cm^−2^ min^−1^, nearly doubling compared to the condition without H_2_O [98]. The underlying mechanism, shown in Figure 5g, involves H_2_O releasing OH^−^, which then transfers to the feed side, where it reacts with CO_2_ to form CO_3_^2−^ [56]. As the CO_3_^2−^ concentration increases, CO_2_ permeation is enhanced, providing an additional permeation pathway. The reaction steps are shown in the following equation:

(5) $$H_{2}O+{{CO}_{3}}^{2-}={{HCO}_{3}}^{-}+OH^{-}$$

(6) $${{HCO}_{3}}^{-}=CO_{2}+OH^{-}$$

Chen et al. investigated the effects of an SDC-MC dual-phase membrane in a H_2_S atmosphere. They found that the introduction of H_2_S led to the formation of Ce_2_O_2_S on the feed side of the dual-phase membrane [99]. Due to the reduced O^2−^ conductivity of SDC, the CO_2_ permeation flux also decreased, as shown in Figure 5h. These findings suggest that gas permeation is influenced not only by the material but also by various physical and chemical factors. Developing membranes with excellent structural integrity and resistance to reducing gasses is of practical significance. To improve permeability, enhancing the O^2−^ conductivity of the solid matrix is effective. Conventional methods include doping, incorporating fluorite phases, and modifying surfaces. In terms of stability, the most important factor is improving the wettability between the ceramic phase and MC. Under long-term experimental conditions, reducing the loss of carbonates is crucial. The physical factors mentioned earlier must all be considered.

**Figure 6:**
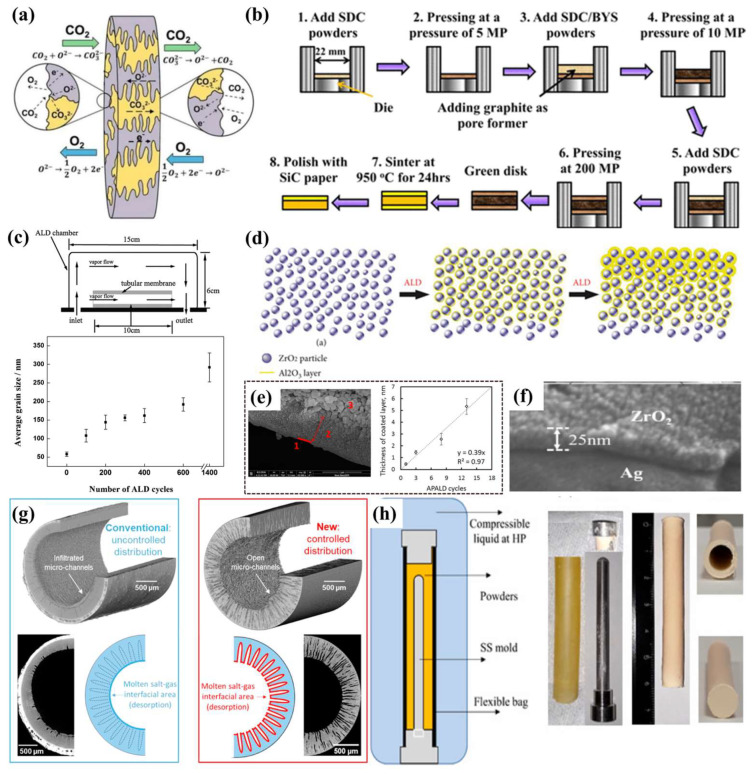
(**a**) Illustration of CO_2_ permeation through a dense mixed ionic–electronic-conducting (MIEC) carbonate dual-phase membrane [100]. (**b**) Schematic drawing of preparation of thin SDC layers on SDC/BYS supports by co-pressing method [101]. (**c**) Schematic diagram of the ALD chamber and the relationship between the surface grain size of the selective layer and the number of ALD cycles. (**d**) Schematic diagram of pore size tailoring of ceramic membranes of sintered nanoparticles by ALD. (**e**) High-magnification SEM images of the layer and the correlation between the thickness of the layer and the number of ALD cycles. (**f**) Microstructure of a porous Ag matrix overcoated with ZrO_2_. (**g**) Schematic diagram of conventional infiltration method and new two-step coating method [71]. (**h**) Schematic representation for making dead-end tubes by CIP method [67].

For the MOCC membranes, different synthesis methods can produce CCDP membranes with varying structures, which have significant implications for their practical applications. Wu et al. synthesized disk-shaped membranes through dry pressing and sintering, as shown in Figure 6a.

They prepared Pr_0.6_Sr_0.4_Co_0.2_Fe_0.8_O_3−*δ*_ and SrFe_0.9_Ta_0.1_O_3−*δ*_-mixed conductor membranes and investigated the effects of CO_2_ and O_2_ permeation and reverse permeation on their performance. They found that the CO_2_ permeation is primarily determined by the total conductivity of CO_3_^2−^ and O^2−^, while O_2_ permeation is controlled by the O^2−^ conductivity of the ceramic phase [100]. In the presence of reverse permeation, CO_2_ reverse permeation becomes the dominant factor, significantly affecting O_2_ permeation. After that, Lu et al. used a compression method to fabricate a porous SDC thin-layer asymmetric support on an SDC-BYS substrate, as presented in Figure 6b. The green Disk consists of three layers: two porous SDC layers at the ends, with a dense BYS-MC layer in the middle. After sintering at 950 °C for 24 h, one of the porous layers was polished away using SiC paper, resulting in the final SDC-BYS asymmetric membrane [101]. The SDC porous layer has a thickness of 150 µm and is impregnated with MC. Under 700 °C, the CO_2_ permeation flux was 6.55 × 10^−3^ mol s^−1^ m^−2^, and stable operation was maintained for 160 h. Also, Zhang et al. synthesized SDC-NiO membranes using co-precipitation and sacrificial template methods, with an SDC ratio ranging from 70:30 to 50:50, and NiO serving as the sacrificial template. It is a sponge-like microstructure, with interconnected pores [102]. Mercury intrusion porosimetry tested the pore diameter as ~600 nm, with a porosity of 50.2%. The membrane exhibits significant CO_2_ permeation properties, with permeation ranging from 0.26 to 1.84 mL cm^−2^ min^−1^ at 700 °C for 30%MC-50%MC. Its CO_2_ flux density is two orders of magnitude higher than that reported for similar systems in the literature. Moreover, Li et al. used atomic layer deposition (ALD) to deposit an Al_2_O_3_ layer onto a 50 nm ceramic microfiltration membrane. By varying the thickness of the Al_2_O_3_ deposit, they created porous ultrathin selective layers with different gradients (Figure 6c,d). Figure 6e exhibits high resolution SEM images of the above sample. In each sample, a newly formed layer including a dense sublayer and a transition sublayer can be detected. The filtration experiments show that ceramic membranes have a decreasing PWF but a rising BSA retention with increasing ALD cycles [103]. As shown in Figure 6e, the separation layer of the received membrane is located at the inner surface of the tubular membrane. The thickness of the deposited layer grows linearly with the increment of coating cycles [104]. Zhang et al. also used ALD to coat a nanoscale ZrO_2_ layer on a Ag substrate. At 850°C, the CO_2_ permeation flux reached 0.9 mL cm^−2^ min^−1^, and long-term stability tests showed that ZrO_2_ improved the membrane’s stability (Figure 6f) [105]. These results highlight the significant potential of ALD technology in membrane separation applications.

Ceramic hollow-fiber membranes with a high surface-area-to-volume ratio have garnered widespread attention in recent years. The loading of carbonate in dual-phase membranes significantly affects CO_2_ permeation, and the high filling density of hollow-fiber membranes effectively meets this requirement [106]. Figure 6g shows that the membrane has a tubular outer structure with conical microchannels inside, allowing molten carbonate to impregnate the microchannels [71]. The hollow-fiber support is typically synthesized using the phase inversion method, where the mixture is extruded through an inner needle, and the external coagulant solidifies the fiber for more than 24 h. Zhuang et al. developed La_0.6_Sr_0.4_Co_0.2_Fe_0.8_O_3−*δ*_ (LSCF) perovskite hollow fibers to serve as porous supports, testing their performance at temperatures ranging from 500 to 900 °C [107]. At 900 °C, the CO_2_ permeation flux reached 1.0 mL cm^−2^ min^−1^. Wu et al. used Nd-doped CeO_2_ as a ceramic hollow-fiber support layer. Nd-doped CeO_2_ (NDC) not only exhibits good bending strength but also demonstrates excellent oxygen-ion conductivity with increasing temperature [68]. At a temperature of 900 °C and using a gas mixture of 50% CO_2_ and 50% N_2_, the permeation flux achieved was 5.08 mL cm^−2^ min^−1^, maintaining stable performance for a duration of 120 h. Additionally, materials such as YSZ, SrFe_0.8_Nb_0.2_O_3−*δ*_ (SFN), and SDC are also commonly used in hollow-fiber membranes [108,109,110].

The synthesis method for tubular membranes is like that of hollow-fiber membranes, with a thick, porous support layer on the exterior and a thin carbonate layer on the inner tube. Similarly, the SDC is a common component in tubular membranes. Dong et al. used centrifugal casting to prepare tubular SDC–carbonate dual-phase membranes and proposed the formation of an asymmetric tubular structure with BYS [111]. The CO_2_ permeation flux reached 1.56 mL cm^−2^ min^−1^ at 900 °C. A comparison of the performance between asymmetric and symmetric membranes revealed that at 800 °C, the asymmetric membranes exhibited a flux that was 3.6 times greater than that of the symmetric membranes. This difference was mainly attributed to variations in membrane thickness and microstructure. Following CO_2_ separation tests conducted under elevated feed pressure and temperature, the structure, morphology, and gas tightness of the SDC-MC membrane showed no alterations (Figure 6h) [67]. This suggests that the SDC-MC membrane demonstrates significant stability in applications involving high-temperature, high-pressure separation, and chemical reactions. In summary, the synthesis method of ceramic support for the ceramic–carbonate membrane can change the microstructure of the membrane and affect its CO_2_ permeation behavior.

### 3.2. MECCs-Based Materials’ Microstructure Engineering

**Figure 7:**
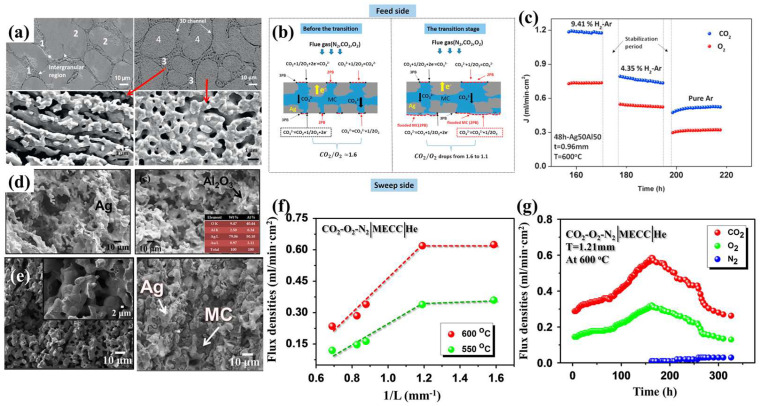
(**a**) Microstructures of Ag50Al50 and 48 h-Ag50Al50 [115]. (**b**) Schematic illustration of the proposed bi-pathway transport mechanism [113]. (**c**) The effect of H_2_ concentration in the sweep gas on CO_2_ and O_2_ flux densities [115]. (**d**) The microstructure of a porous Ag matrix and a porous Ag matrix coated with 5% Al_2_O_3_ colloidal [117]. (**e**) A schematic illustration of the self-forming NiO-MECC membrane. (**f**) The CO_2_ densities of the MECC membrane as a function of the reciprocal of thickness at 550 °C and 600 °C. (**g**) The CO_2_/O_2_ flux density and selectivity of the NiO–MC membrane measured at 850 °C [118].

In MECC membranes, the ceramic phase acts as an electronic conductor, allowing only electrons to pass through. The first MECC membrane was reported by Lin and his co-authors, using stainless steel (SS) as the porous substrate and electronic conductor [112]. The permeation performance was tested at temperatures ranging from 450 to 750 °C. Below 650 °C, the permeation flux was positively correlated with temperature; however, above 650 °C, CO_2_ permeation decreased with increasing temperature. This may be due to the reaction between SS and MC at high temperatures, leading to poor chemical compatibility. Huang’s research team chose silver (Ag), recognized for its excellent stability, to serve as the electronic conductor for the MECC membrane [113]. The permeation mechanism is illustrated in Figure 7b. Due to the excellent electronic conductivity of Ag, the CO_2_ permeation flux reached 0.82 mL cm^−2^ min^−1^ at 650 °C, five times higher than that of SS. However, when the temperature exceeds 650°C, the stability of Ag-MC decreases due to the loss of MC by evaporation [114]. To further improve the wettability of Ag with MC, Huang et al. fabricated a silver-based porous silver-carbonate dual-phase membrane using an electrochemical dealloying method [115]. The preferred alloy for chemical dealloying was 50%Ag-50%Al, a method commonly employed to prepare nanoporous metals [116]. The microstructure of the alloy and a high-magnification SEM image are shown in Figure 7a. The effect of adding H_2_ to Ar as a sweep gas on the CO_2_ and O_2_ flux densities was investigated. In addition, Figure 7c shows the measured CO_2_ and O_2_ flux densities (*J*_CO2_ and *J*_O2_) over the membrane lifetime at 600°C, using Ar with varying H_2_ concentrations as the sweep gas. The results indicate that the CO_2_ and O_2_ flux densities were highest with 9.41% H_2_-Ar, approximately 1.5 times higher than with 4.35% H_2_ + 95.65%Ar and twice as high as with pure Ar. This confirms that reducing the O_2_ partial pressure can significantly enhance the CO_2_ and O_2_ flux densities. Surface modification with Al_2_O_3_ has been shown to improve the wettability between the ceramic phase and MC.

Additionally, Huang et al. infiltrated 5% and 10% Al_2_O_3_ colloidal solutions into the Ag support under vacuum conditions. SEM analysis revealed a pore size of 15–20 μm, with Al_2_O_3_ successfully coating the surface of the Ag substrate (Figure 7d). High-temperature stability tests showed that after 130 h, the performance retention was 90% [117]. Additionally, factors such as membrane thickness and flow rates on both sides can affect permeation performance. Huang et al. systematically studied the effects of membrane thickness, CO_2_ concentration, and other factors on the permeation and stability of silver-carbonate membranes [118]. The membrane thickness was controlled between 0.7 and 1.6 mm, and the study found that performance decreased rapidly as thickness increased from 0.7 mm to 1.2 mm. Further increasing the thickness resulted in little change in permeation flux, with a thickness of ~0.84 mm (as shown in Figure 7e–g). Although Ag exhibits excellent permeation performance, its high cost restricts its widespread application. Zhang et al. reported a cost-effective, electronically conductive NiO-MC dual-phase membrane, in which NiO and MC in situ form Li_0.4_Ni_1.6_O_2−*δ*_ (LNO), a high electron conductivity phase [119]. The formation of the LNO is achieved via a preactivation process, leading to a gradual increase in gas permeation during the early stages of long-term testing. During the test, the CO_2_/O_2_ ratio remained ~2:1. At 850 °C, the CO_2_ permeance of the NiO-MC membrane can reach 1.0 mL cm^−2^ min^−1^. In the CO_2_ transport process, both the formation of CO^2−^ ions with electrons in the metal phase and the release of gaseous CO_2_ from the feed gas require O_2_. Thus, nickel-carbonate membranes are effective for separating CO_2_ from O^2−^-containing sources. Overall, MECC membranes require high electronic conductivity in the ceramic phase, which limits the research.

### 3.3. MEOCCs-Based CO_2_ Separation Performance Optimized

**Figure 8:**
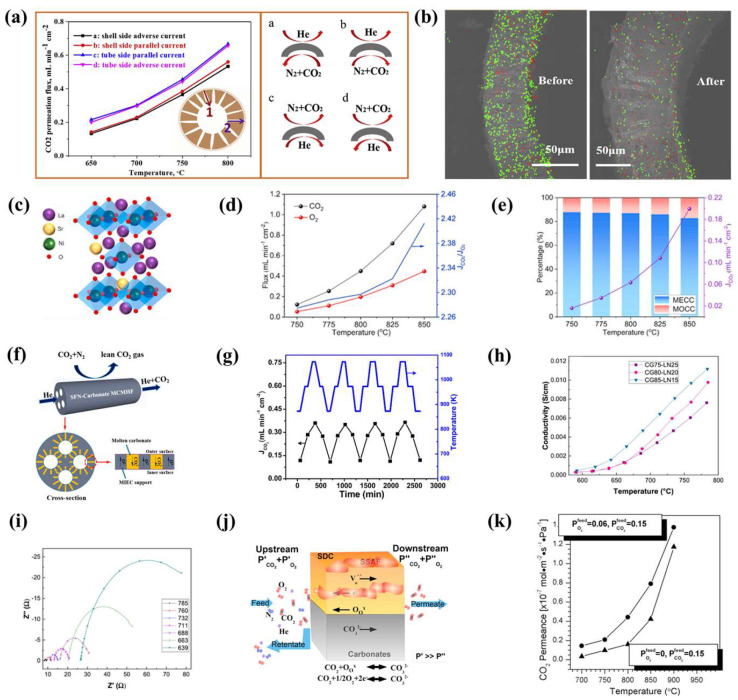
(**a**) Effects of different gas flow directions on CO_2_ permeation in LSCF hollow-fiber membranes and its schematic diagram. (**b**) Elemental mapping images of the cross-section before and after long-term stability testing [90]. (**c**) Structural schematic of La_1.5_Sr_0.5_NiO_4+*δ*_. (**d**) CO_2_ permeation flux at different temperatures [77]. (**e**) CO_2_ flux under the MOCC model and the transmission ratio between MOCC and MECC pathways [122]. (**f**) Schematic diagram of SrFe_0.8_Nb_0.2_O_3−*δ*_ four-channel hollow-fiber membrane. (**g**) Thermal shock resistance [109]. (**h**,**i**) Conductivity of different GDC and LN ratios at varying temperatures and Nyquist plots of CG80-LN20 [123]. (**j**) Permeation mechanism of the SDC-SSFA-MC membrane. (**k**) CO_2_ permeation of the SDC-SSFA-MC membrane [124].

The operating mechanism of the MEOCC membrane involves both the MECC and MOCC modes. Perovskite materials are used as the porous support in MEOCC membrane reactors due to their high ionic and electronic conductivity, which enables operation under both oxygen-rich and oxygen-free conditions. Among these materials, LSCF was initially reported as a molten-carbonate support. Subsequent research has investigated the effects of temperature, membrane thickness, and support porosity on CO_2_ permeation [55,120]. Zhuang et al. fabricated LSCF hollow-fiber membranes using the reverse spinning method and evaluated their CO_2_ permeability and stability [90]. The results indicated that the CO_2_ permeation flux was higher when the shell side acted as the feed side and the lumen side as the sweep side, due to the larger surface area on the shell side and the faster gas flow on the lumen side, as depicted in Figure 8a. Furthermore, after a durability test at 700 °C for 77 h, element mapping of the membrane cross-section (Figure 8b) revealed a decline in performance over time, which was attributed to the loss of the molten-carbonate phase.

Zeng et al. fabricated Mo-doped LSF-MC dual-phase membranes. Although Mo doping reduced the conductivity of the membrane, it improved the wettability of the porous support with molten carbonate, thereby enhancing CO_2_ permeation performance [121]. Furthermore, they further enhanced the contact between the support and the molten carbonate by coating the surface of the LSFM support with Al_2_O_3_, which increased the number of active sites for surface reactions. As shown in Figure 8c, Tong et al. fabricated a La_1.5_Sr_0.5_NiO_4+*δ*_-MC membrane without Fe [77]. Figure 8d illustrates that when simulated flue gas and Ar were used as feed and sweep gasses, respectively, the CO_2_ permeation flux increased from 0.12 to 1.08 mL·cm^−2^·min^−1^, while the O_2_ permeation flux rose from 0.05 to 0.45 mL·cm^−2^·min^−1^ as the temperature increased from 750 to 850 °C. The CO_2_/O_2_ permeation ratio, which exceeded two and steadily increased, indicated that CO_2_ permeation in the simulated flue gas was facilitated by both MECC and MOCC interactions.

As exhibited in Figure 8e, the CO_2_ flux increased from 0.02 to 0.2 mL·cm^−2^·min^−1^ as the temperature rose from 750 to 850 °C, and the MOCC/MECC ratio also increased. Furthermore, the incorporation of fluoride with good chemical stability and relatively high ionic conductivity into the porous support will enhance the permeation property and stability of MEOCC membranes [122]. As illustrated in Figure 8f, Jiang et al. fabricated a SrFe_0.8_Nb_0.2_O_3−*δ*_-MC four-channel hollow-fiber membrane permeated with ternary molten carbonate [109]. They investigated its CO_2_ permeation performance, mechanical properties, and thermal shock resistance. Revealing that the infiltration of molten carbonate into the porous fiber substrate enhanced its mechanical strength, while the CO_2_ permeation flux increased with rising temperature and CO_2_ partial pressure difference. Furthermore, the membrane maintained a stable CO_2_ permeation flux during repeated heating and cooling cycles at 873, 973, and 1073 K, demonstrating excellent thermal stability (Figure 8g).

Very recently, González-Varela et al. prepared GDC-LN-MC ternary membranes with varying ratios [123]. Conductivity and impedance tests on these three samples revealed that the material’s conductivity increased with both the fluorite phase content and temperature (Figure 8h,i). However, a high fluorite phase content resulted in oxygen-ion enrichment on the surface, which subsequently reduced CO_2_ adsorption. Consequently, the GDC80-LN20 membrane exhibited superior permeation performance compared to GDC85-LN15. Meanwhile, O. Ovalle-Encinia et al. synthesized SDC-SSFA-MC ternary membranes using both single-step and two-step methods [124]. They discovered that only the membrane fabricated from the dual-phase powder synthesized via the single-step method exhibited good thermal and chemical stability. The CO_2_ permeation mechanism of the membrane is illustrated in Figure 8j. Figure 8k shows the CO_2_ permeation flux of the MEOCC membrane under varying temperatures and atmospheric conditions. The CO_2_ permeation flux increases with the temperature. Moreover, under oxygen-rich conditions, the CO_2_ permeation flux is higher compared to oxygen-free conditions due to the incorporation of the MECC mode. In general, MEOCC membranes exhibit higher CO_2_ flux compared to MOCC and MECC membranes. However, the presence of O_2_ on the feed side is crucial for achieving high flux, leading to a mixture of O_2_ and CO_2_ on the permeate side. Additional O_2_ separation processes are necessary.

**Table 2:** Performance of MOCC membranes in CO_2_ permeation and long-term stability tests.

Material	Configuration	Thickness (mm)	Test Condition			Flux	Stability Test			Ref.
			Feed Side	Sweep Side	T (°C)		T (°C)	Time (h)	*J*_CO2_	
SDC (Li/Na)	Tubular	1	CO_2_/N_2_	He	710	0.46 mL cm^−2^ min^−1^	-	-	-	[67]
NDC (Li/Na)	Hollow fiber	0.1~0.15	CO_2_/N_2_	Ar	900	5.08 mL cm^−2^ min^−1^	700	120	~0.36 mL cm^−2^ min^−1^	[68]
GDC−82 (Li/Na)	Disk	1	CO_2_/N_2_	Ar	850	0.77 mL cm^−2^ min^−1^	850	200	0.77 mL cm^−2^ min^−1^	[79]
GDC−91-ε20 (Li/Na)	Disk	1	CO_2_/N_2_	Ar	650	0.125 mL cm^−2^ min^−1^	650	250	~0.13 mL cm^−2^ min^−1^	[81]
GDC (Li/Na)	Disk	0.75	CO_2_/He/N_2_	Ar	800	1.38 mL cm^−2^ min^−1^	-	-	-	[83]
Al_2_O_3_-ScCeSZ (Li/Na)	Disk	1	CO_2_/N_2_	Ar	650	~1.0 mL cm^−2^ min^−1^	650	90	1.5 mL cm^−2^ min^−1^	[56]
SDC (Li/Na)	Disk	1.18	CH_4_/ CO_2_/N_2_	-	700	0.11 mL cm^−2^ min^−1^	650	100	>0.12 mL cm^−2^ min^−1^	[88]
SDC (Li/Na)	Tubular	0.9	CO_2_/N_2_	He	600	0.55 × 10^−3^ mol m^−2^ s^−1^	-	-	-	[89]
Ni/Ca-SDC (Li/Na)	Tubular	0.9	CO_2_/N_2_	H_2_/He	600	0.76 × 10^−3^ mol m^−2^ s^−1^	500	32	~0.3 mol m^−2^ s^−1^	
SDC (Li/Na)	Disk	0.8	CO_2_/H_2_/N_2_	-	750	0.87 mL cm^−2^ min^−1^	-	-	-	[99]
SDC/SDC-BYS (Li/Na/K)	Disk	0.15	CO_2_/N_2_	He	700	6.55 × 10^−3^ mol m^−2^ s^−1^	700	160	6.55 × 10^−3^ mol m^−2^ s^−1^	[101]
BZY-20C (Li/Na)	Disk	0.8	CO_2_/N_2_	Ar	750	0.52 mL cm^−2^ min^−1^	-	-	-	[98]
			CO_2_/N_2_	3%H_2_O/Ar	750	0.62 mL cm^−2^ min^−1^	650	250	0.45 mL cm^−2^ min^−1^	
YSZ (Li/Na/K)	Hollow fiber	0.3	CO_2_/N_2_	He	950	0.22 mL cm^−2^ min^−1^	-	-	-	[108]
SDC (Li/Na)	Hollow fiber	0.1–0.15	CO_2_/N_2_	He	700	4.78 mL cm^−2^ min^−1^	600	85	0.95 mL cm^−2^ min^−1^	[110]
			CO_2_/H_2_/N_2_	He	700	5.46 mL cm^−2^ min^−1^				
			CO_2_/O_2_/N_2_	He	700	1.79 mL cm^−2^ min^−1^				
SDC/SDC-BYS (Li/Na/K)	Tubular	0.15	CO_2_/N_2_	He	900	1.56 mL cm^−2^ min^−1^				[111]
SDC (Li/Na/K)	Tubular	1.5	CO_2_/N_2_	He	900	~0.5 mL cm^−2^ min^−1^				

**Table 3:** CO_2_ permeation performance and long-term stability of different MECC membranes.

Material	Configuration	Thickness (mm)	Test Condition			Flux	Stability Test			Ref.
			Feed Side	Sweep Side	T (°C)		T (°C)	Time (h)	*J*_CO2_	
SS (Li/Na/K)	Disk	1.57	CO_2_/O_2_	-	650	2.5 × 10^−8^ mol s^−1^ m^−2^ Pa^−1^	-	-	-	[112]
Ag (Li/K)	Disk	1.67	CO_2_/O_2_/N_2_	He	650	0.82 mL cm^−2^ min^−1^	650	20	0.82 mL cm^−2^ min^−1^	[113]
Ag (Li/Na)	Disk	1.02	CO_2_/O_2_/N_2_	Ar/H_2_	700	1.30 mL cm^−2^ min^−1^	600	900	~1 mL cm^−2^ min^−1^	[115]
Ag (Li/Na)	Disk	0.91	CO_2_/O_2_/N_2_	Ar/H_2_	650	0.89 mL cm^−2^ min^−1^	600	400	~1.15 mL cm^−2^ min^−1^	[116]
Al_2_O_3_-coated Ag (Li/K)	Disk	1.23	CO_2_/O_2_/N_2_	He	650	0.39 mL cm^−2^ min^−1^	650	130	0.32 mL cm^−2^ min^−1^	[117]
Al_2_O_3_-coated Ag (Li/K)	Disk	1.14	CO_2_/O_2_/N_2_	He	650	0.47 mL cm^−2^ min^−1^	600	320	0.28 mL cm^−2^ min^−1^	[118]
NiO (Li/Na)	Disk	1.20	CO_2_/O_2_/N_2_	He	850	1.0 mL cm^−2^ min^−1^	850	310	1.0 mL cm^−2^ min^−1^	[119]
ALD-ZrO_2_-Ag (Li/Na)	Disk		CO_2_/O_2_/N_2_	Ar	850	0.9 mL cm^−2^ min^−1^	800	40	>0.8 mL cm^−2^ min^−1^	[105]

**Table 4:** Performance of MEOCC membranes in CO_2_ permeation and long-term stability tests.

Material	Configuration	Thickness (mm)	Test Condition			Flux	Stability Test			Ref.
			Feed Side	Sweep Side	T (°C)		T (°C)	Time (h)	*J*_CO2_	
LSCF (Li/Na/K)	Disk	0.76	CO_2_/O_2_/N_2_	He	850	0.256 mL cm^−2^ min^−1^	-	-	-	[120]
LSFMo (Li/Na)	Disk	1	CO_2_/O_2_/N_2_	He	800	2.69 × 10^−3^ mol m^−2^ s^−1^	750	50	1.54 × 10^−3^ mol m^−2^ s^−1^	[121]
LSN (Li/Na)	Disk	0.8	CO_2_/O_2_/N_2_	Ar	850	1.08 mL cm^−2^ min^−1^	850	120	5.2 × 10^−7^ mol s^−1^ m^−2^ Pa^−1^	[77]
Au-Pd-coated SDC-SSAF- (Li/Na/K)	Disk	0.9	CO_2_/He/O_2_/N_2_	N_2_	900	1.72 × 10^−7^ mol s^−1^ m^−2^ Pa^−1^	-	-	-	[122]
SFNb (Li/Na/K)	Hollow fiber	0.22	CO_2_/N_2_	He	850	0.64 mL cm^−2^ min^−1^	700	200	0.31 mL cm^−2^ min^−1^	[109]
CG80-LN20 (Li/Na/K)	Disk	0.94	CO_2_/He/N_2_	Ar	900	4.34 × 10^−7^ mol s^−1^ m^−2^ Pa^−1^	-	-	-	[123]
SDC-SSAF (Li/Na/K)	Disk	-	CO_2_/He/O_2_	N_2_	900	1.7 × 10^−7^ mol s^−1^ m^−2^ Pa^−1^	-	-	-	[124]
SFTa (Li/Na/K)	Disk	1.5	CO_2_/He	Air	950	0.165 mL cm^−2^ min^−1^	-	-	-	[100]

## 4. The Potential Applications of Membrane Reactors

A CCDP membrane, constituted by a mixed ionic–electronic-conducting (MIEC) oxide phase and a molten-carbonate phase, enables the selective separation of CO_2_ within the temperature range from 400 to 1000 °C. Under the partial pressure disparity of CO_2_ and O_2_, the solid oxide phase serves as a support for the molten-carbonate phase, facilitating the diffusion of carbonate ions, electrons, and oxide ions across the membrane. Recently, the proposal for carbon peaking and carbon neutrality goals has drawn significant attention to the capture and utilization of CO_2_. Two major CO_2_ conversion technologies are the dry reforming of methane (DRM) and reverse water–gas shift (RWGS), both of which are highly endothermic processes. The application of CO_2_ permeation membranes enables simultaneous CO_2_ capture and conversion within the same reactor, simplifying the system while reducing energy consumption and costs. In this section, we review various studies on CO_2_ permeation membrane reactors for CO_2_ capture and conversion.

**Figure 9:**
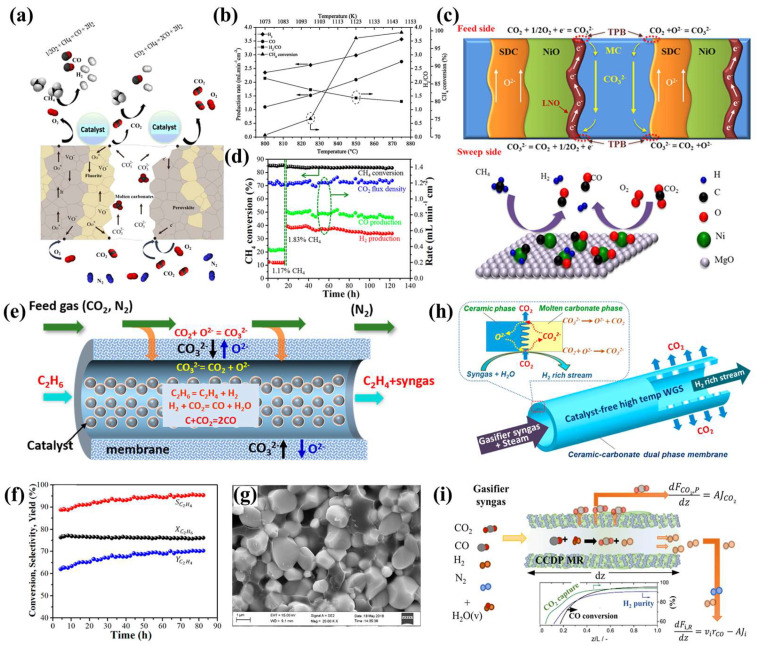
(**a**) Schematic diagram of the CP-PSFC-MC membrane coupled with DRM. (**b**) DRM performance of the CP-PSFC-MC membrane at different temperatures [40]. (**c**) Schematic diagram of the SDC-NiO-MC membrane used in DRM. (**d**) Long-term stability test of the SDC-NiO-MC membrane coupled with DRM [125]. (**e**–**g**) Ethane-to-ethylene conversion using a GDC-MC membrane reactor: (**e**) mechanism diagram, (**f**) long-term stability, and (**g**) SEM image of the surface after testing [126]. (**h**) SDC-MC tubular membrane for hydrogen production via the WSG mechanism [127]. (**i**) Numerical simulation of ceramic–MC membrane for hydrogen production via WSG and the influence of membrane thickness [128].

As shown in Figure 9a, J.A. Fabián-Anguiano et al. developed a CO_2_ separation membrane by loading a porous Ce_0.9_Pr_0.1_O_2−*δ*_-Pr_0.6_Sr_0.4_Fe_0.5_Co_0.5_O_3−*δ*_ (CP-PSFC) support with ternary molten carbonates and coupled it with DRM using 10 wt.% Ni/γ-Al_2_O_3_ as the catalyst [40]. Figure 9b shows that under DRM conditions (875 °C), the membrane reactor achieved a CH_4_ conversion rate of 99% and a syngas yield of 6.25 mL·cm^−2^·min^−1^. The observed decrease in the H_2_/CO ratio was attributed to methane decomposition. As exhibited in Figure 9c, Zhang et al. prepared an SDC-NiO-MC composite membrane using a coprecipitation method. At elevated temperatures, the NiO phase reacts with Li_2_CO_3_ in the MC phase to form the electron-conducting LiNiO_2_ phase, which increases the CO_2_ permeation pathway and enhances CO_2_ permeation during the early stages of the reaction [125]. When coupled with the DRM reaction using 1 wt.% Pt-Ni_0.2_Mg_0.8_O as the catalyst, the membrane achieved a CH_4_ conversion rate of 85% and a syngas yield of 7.4 mL·cm^−2^·min^−1^ at 850 °C, demonstrating stable operation for over 130 h, as displayed in Figure 9d.

In 2019, Zhang et al. prepared a GDC-MC tubular membrane that was internally filled with 5 wt.% Cr_2_O_3_/ZSM-5 catalyst (Figure 9e) [126]. This setup facilitated the coupling of CO_2_ permeation with ethane thermal dehydrogenation (TDHE). The CO_2_ permeating through the GDC-MC dual-phase membrane undergoes a RWGS reaction with hydrogen generated during the TDHE process, thereby enhancing ethylene production. The membrane reactor demonstrated stable operation for 80 h at 800 °C, with no carbon deposition observed, as depicted in Figure 9f,g. Moreover, Dong and Lin prepared an SDC-MC dual-phase membrane using a centrifugal casting method, which couples CO_2_ separation with the WGS reaction (as shown in Figure 9h) [127]. The membrane reactor effectively separates CO_2_ from the WGS products, thereby enhancing hydrogen production via the WGS reaction. In 2021, Meng et al. also investigated the effects of temperature, feed-side partial pressure, sweep gas flow rate, membrane thickness on CO_2_ capture capacity, and WGS performance by numerical simulations (as illustrated in Figure 9i) [128].

In addition to the studies, Zhang et al. prepared a GDC-MC membrane loaded with LaNi_0.6_Fe_0.4_O_3−*δ*_(LNF) and Ni-MgO-1 wt.% Pt catalysts and coupled it with DRM, comparing the performance of the two catalysts [129]. The results indicated that the LNF catalyst performed slightly worse than Ni-MgO-1 wt.% Pt, exhibiting a slower activation process at the beginning of the test, possibly due to the dissolution of Ni nanoparticles. After that, Chen et al. prepared a LN-SDC-MC membrane and coupled it with the RWGS reaction for hydrogen production using LN and La_0.9_Ce_0.1_NiO_3−*δ*_ (LCN) as catalysts [38]. The higher oxygen vacancy concentration and more uniform dispersion of LCN made it more favorable for promoting the RWGS reaction. Pang et al. prepared an SDC-MC tubular membrane filled with a 15 wt.% Ni-5 wt.% M/SDC (M = Ca, Mg, Ba) catalyst to facilitate CO_2_ methanation, thereby enhancing CO_2_ capture [89]. Tong et al. prepared a La_1.5_Sr_0.5_O_4+*δ*_-MC dual-phase tubular membrane loaded with a La-Sr/CaO catalyst for the oxidative coupling of methane (OCM) to produce ethylene and ethane. At 850 °C, the membrane reactor achieved a CH_4_ conversion rate of 28.0%, a selectivity of 43.8%, and a yield of 12.3%, outperforming a fixed-bed reactor with stable operation for over 100 h [130]. The membrane reactor achieved methane selectivity and CO_2_ conversion rates exceeding 95%. In summary, researchers have verified the potential applications of CO_2_ permeation membranes in DRM [78,131,132,133,134], WGS/SRM [39], RWGS, TDHE, and OCM [135], as well as CO_2_ methanation. However, further efforts are required in membrane material design and catalyst development to address challenges such as variable operating conditions (e.g., temperature, atmosphere), product yield, and selectivity.

## 5. Conclusion and Perspectives

### 5.1. Concluding Remarks

This review offers a comparison among diverse CO_2_ capture routes and technologies, emphasizing the suitability of the CCDP membrane reactors for post-combustion CO_2_ capture on account of their outstanding high-temperature stability. This article categorizes three mechanisms for CO_2_ separation through CCDP membranes. The MECC membrane enables CO_2_ permeation merely in the presence of oxygen, yet it confronts challenges like high-temperature sintering and poor wettability with molten carbonates. In contrast, the MOCC membrane functions without the requirement of oxygen, although the oxygen-ion conductivity of the porous support material restricts CO_2_ permeation. Modifying the membrane surface and microstructure to enhance the wettability between the porous support and molten carbonates can notably augment CO_2_ permeability. Factors such as the operating temperature, membrane thickness, and configuration have an impact on the surface reaction rate and bulk diffusion process within the membrane reactor. Additionally, alterations in the composition, concentration, and flow rate of the feed or sweep gas can influence CO_2_ permeation. Finally, several application scenarios based on CCDP membrane reactors are deliberated. By employing CH_4_ as a sweep gas, CO_2_ and O_2_ captured by the same membrane reactor can undergo DRM and RWGS reactions to produce syngas. Alternatively, a membrane reactor is capable of separating CO_2_ from the products of the WGS process, thereby generating high-purity H_2_. Although the feasibility of membrane reactors for the capture and conversion of CO_2_ has been experimentally verified and demonstrates considerable potential, further comprehensive exploration in materials, the design of membrane reactors, as well as operation and maintenance are indispensable.

### 5.2. Future Perspectives

The membrane separation technology of CO_2_ demonstrates a significant application prospect in the domain of future clean power generation. Its application can not only effectively enhance the efficiency of energy conversion and generation but also plays a positive role in reducing the adverse impacts on the environment through the capture and utilization of CO_2_. This technology is anticipated to be appealing to large CO_2_ emission sites that lack the capacity for using CO_2_ in enhanced oil recovery or geological storage. These sites are frequently under intense pressure to reduce emissions, yet it is challenging to directly utilize or store substantial amounts of CO_2_ emissions due to technical and economic constraints. The membrane separation technology enables economically feasible CO_2_ capture and treatment, offering a flexible and scalable solution for these sites.

From the perspective of practical application, given the low maturity of the technology, the future of membrane separation technology should consider the following aspects:

(1) Develop novel membrane materials and corresponding production processes to fabricate CO_2_ separation membranes with excellent performance. The membranes for CO_2_ separation should feature high permeability selectivity and stability. Currently, only a fraction of membranes has demonstrated high performance and long-term stability under practical circumstances, which fails to satisfy the requirements of practical application scenarios. In the future, more membrane materials need to be developed to regulate and optimize the structure of membrane materials from the molecular scale, thereby enhancing their separation performance and stability.

(2) Fully take into account the gaseous flow state and mass transfer characteristics for the development of new membrane components. Membrane components with conventional structures are incapable of meeting the demand for the efficient separation of CO_2_ from flue gas. A calculation model of the internal pressure drop of membrane components ought to be established based on the performance indexes and operating conditions of membrane materials, to analyze the internal flow field and optimize the structure of membrane components. Meanwhile, the innovation and optimization of synthesis methods should be carried out to develop new synthesis strategies for manufacturing porous solid substrates with small and uniform pores while reducing costs.

(3) The subsequent research domain lies in the direct application of the catalyst onto the membrane surface. The incorporation of the catalyst can conspicuously enhance the efficiency of membrane separation. For instance, the addition of a specific catalyst within the membrane material can facilitate the diffusion and penetration of carbon dioxide molecules within the membrane, thereby elevating the separation efficiency. Additionally, the catalyst can also lower the activation energy of the reaction, rendering the membrane separation process more facile. With the continuous advancement of technology, it is of great significance to develop novel catalytic systems to enhance the separation efficiency and selectivity of CO_2_ and promote the conversion of CO_2_.

## Data Availability

Data sharing is not applicable.
